# Gestational Age and Birth Weight as Predictors of NICU Admission Probability and Length of Stay in a Jordanian Neonatal Cohort

**DOI:** 10.7759/cureus.115997

**Published:** 2026-09-09

**Authors:** Mai N Almasaeed, Rawand Alqtaishat, Abdullah A Alnawaflh, Rana S Mashagbeh, Baraa M Al Falahat

**Affiliations:** 1 Pediatrics and Child Health, Jordanian Royal Medical Services, Amman, JOR; 2 Pediatrics and Neonatology, Jordanian Royal Medical Services, Amman, JOR; 3 Pediatric Gastroenterology, Jordanian Royal Medical Services, Amman, JOR

**Keywords:** birth weight, extended hospitalization stay, gestational age, length of stay, neonatal intensive care unit (nicu), parity

## Abstract

Background

Predicting the length of stay (LOS) in the neonatal intensive care unit (NICU) is crucial for resource allocation and clinical management.

Objective

This study aimed to develop and validate a simple, practical predictive model using gestational age (GA) and birth body weight (BBW) in a Jordanian cohort.

Methods

This retrospective study analyzed 218 neonates admitted to the NICU between January 2022 and December 2023. Binary logistic regression was employed to predict prolonged LOS (≥7 days), multiple linear regression to estimate actual LOS, Cox proportional hazards regression to model time-to-discharge, and receiver operating characteristic (ROC) analysis to assess discriminative capacity. Subgroup analyses were performed using GA categories and small for gestational age (SGA) / appropriate for gestational age (AGA) / large for gestational age (LGA) classifications. Temporal validation was conducted on an independent 2024 cohort (n = 87).

Results

The cohort's mean GA was 33.15 ± 2.81 weeks, mean BBW was 2.61 ± 0.52 kg, and median LOS was seven days (IQR: 5-11 days). The multiple linear regression model was highly significant (R² = 0.71, p < 0.001): LOS = 34.12 - (0.59 × GA) - (3.85 × BBW). BBW demonstrated superior predictive performance (AUC = 0.81) compared to GA (AUC = 0.75). SGA infants had significantly longer LOS (11.8 ± 4.6 days) compared to AGA infants (7.9 ± 3.8 days, p < 0.001), with 3.4-fold higher odds of prolonged stay. Temporal validation confirmed model stability (validation R² = 0.694, AUC = 0.821).

Conclusions

GA and BBW are strong, quantifiable predictors of NICU LOS. The developed regression models provide a practical bedside tool for early risk stratification, potentially improving resource planning and targeted care for neonates at a high risk of extended hospitalization in Jordan.

## Introduction

Neonatal intensive care units (NICUs) serve the sickest and most premature infants, making them a crucial aspect of contemporary perinatal medicine [1]. These highly specialized facilities use cutting-edge technology and a multidisciplinary team to treat a wide range of conditions related to organ development, birth deformities, and complications arising from birth [2]. Accurate prediction of a newborn's length of stay (LOS) in the NICU is essential for improving clinical management, helping families prepare, maximizing scarce and costly resources, and achieving better long-term patient outcomes [3,4].

The scientific literature has repeatedly shown that gestational age (GA) and birth body weight (BBW) are the two most critical biological parameters affecting infant health and NICU utilization. GA, a marker of fetal maturity, is adversely associated with respiratory distress syndrome, intraventricular hemorrhage, and necrotizing enterocolitis [5,6]. BBW also indicates intrauterine growth and nutritional status. Classifications such as “extremely low birth weight” (<1,500 g) significantly affect the duration and intensity of medical treatment. It is widely established that decreased GA is associated with reduced BBW, and together they constitute a precarious clinical state that may require long-term specialized care [7,8]. The association between BBW and neonatal outcomes, including NICU LOS, has been extensively documented in the global literature, confirming its role as a consistent and powerful predictor across diverse healthcare settings [7-10].

Even while this basic relationship is widely recognized, it remains challenging to integrate this knowledge into precise, quantitative, and easy-to-use bedside prediction tools, particularly in specific healthcare contexts [9]. Recognizing that NICU LOS is influenced by numerous clinical factors beyond GA and BBW, including respiratory disease, sepsis, feeding problems, surgery, congenital anomalies, and other clinical morbidities, we aimed to develop a simple baseline model that could serve as an initial risk stratification tool using only the two most universally available and objectively measured admission parameters. By “parsimonious model,” we refer to a prediction tool that balances simplicity and clinical utility by employing only the most accessible and objectively measured predictors, while acknowledging that comprehensive models may require additional covariates not universally available at admission. This approach prioritizes ease of use at the bedside, while acknowledging that comprehensive clinical prediction requires additional covariates. Many studies have verified the association, but they have not generated regression equations or survival models that clinicians can use to assess each patient's risk [10]. This gap is particularly evident in middle-income countries such as Jordan, where NICU bed utilization is crucial for both efficiency and equitable access to essential treatment [11]. Identifying the infants most likely to remain in the hospital for a long period as soon as possible might help physicians make decisions, speed up treatment, and enable families and healthcare professionals to make more realistic plans [12].

Survival analysis techniques such as Cox proportional hazards regression can better understand "time-to-event" [13]. This technique predicts hospital discharge across the entire stay, providing dynamic information that a linear model cannot provide. Understanding GA and BBW discharge rates helps manage patient flow and expectations.

The goal of this research was to address these significant gaps by analyzing a current sample of Jordanian infants. We designed and tested robust statistical models that go beyond discovering an association. While the predictive role of BBW is well-established, our study contributes by providing a validated, quantified predictive model tailored to the Jordanian context, offering effect estimates and regression equations that can be readily applied at the bedside. Our primary objective was to develop and validate a simple baseline predictive model that utilizes GA and BBW as readily available predictors of NICU LOS. Our secondary objectives were to (1) determine the probability of prolonged NICU stay (≥7 days) using binary logistic regression, (2) construct a multiple linear regression model for direct estimation of NICU LOS in days, (3) evaluate the dynamic hazard of discharge over time using Cox proportional hazards regression, and (4) examine the impact of clinically relevant GA categories and SGA classification on NICU LOS outcomes.

## Materials and methods

Ethics statement

This study received ethical approval from the Institutional Review Board (IRB) of the Jordanian Royal Medical Services (Reference Number: 24_16/2024) on November 5, 2024. Final authorization for data dissemination and publication was provided by the Jordanian Royal Medical Services Educational and Training Directorate on December 18, 2024. The IRB granted a formal waiver for individual informed consent due to the retrospective, anonymized, and observational nature of the research. The study was conducted in accordance with the ethical principles outlined in the Declaration of Helsinki.

Study design and setting

A retrospective cohort study was conducted at the Neonatal Intensive Care Unit of the Queen Rania Abdullah Hospital for Children, a tertiary care referral center within the King Hussein Medical Centre, Royal Medical Services, Amman, Jordan. The hospital is the first and only specialized pediatric institution in Jordan, serving as a national referral center for complex pediatric and neonatal cases across 22 subspecialties, with a neonatal unit equipped with 14 incubators and 18 intensive care beds [1]. The hospital manages approximately 700 patients daily and performs over 6,000 surgical procedures annually [1]. The study was designed and reported in accordance with the STROBE guidelines for observational studies [14]. The study period spanned from January 1, 2022, to December 31, 2023.

Study population and eligibility criteria

The initial source population comprised all neonates admitted to the NICU during the study period (N = 245). The final analytical cohort included 218 neonates after applying the eligibility criteria. Table 1 presents the inclusion and exclusion criteria for study participation.

**Table 1:** Inclusion and exclusion criteria This table presents the inclusion and exclusion criteria for study participation. Criteria are listed separately for inclusion and exclusion with appropriate descriptions. LOS, length of stay; NICU, neonatal intensive care unit

Criteria
Inclusion criteria
Admission to the NICU for any cause
Availability of complete data for primary variables (gestational age, birth body weight, and NICU LOS)
Exclusion criteria
NICU stay of less than 24 hours
Major congenital anomalies or syndromes known to profoundly and independently affect LOS
Neonates who died during the admission
Records with missing data for primary study variables exceeding 5%

Data sources and variables

Data were meticulously extracted from two primary sources: the hospital's comprehensive electronic medical records system and physical patient files for cross-verification. A standardized, pre-piloted data extraction form was used to ensure consistency in the data collection process.

The primary variables collected were as follows:

GA: measured in completed weeks, primarily determined from first-trimester obstetric ultrasound or, if unavailable, from the mother's last menstrual period. For subgroup analyses, GA was categorized according to WHO classification: very preterm (<32 weeks), moderate preterm (32-33 weeks), late preterm (34-36 weeks), and term (≥37 weeks).

BBW: measured in kilograms (kg) immediately after birth using calibrated digital scales. For specific analyses, BBW was categorized as low birth weight (<2.5 kg) and normal birth weight (2.5-4.0 kg). Additionally, infants were classified as small for gestational age (SGA, <10th percentile), appropriate for gestational age (AGA, 10th-90th percentile), or large for gestational age (LGA, >90th percentile) using INTERGROWTH-21st standards.

NICU LOS: defined as the total number of calendar days from the date and time of NICU admission to the date and time of transfer out of the intensive care unit to a lower-acuity setting (step-down unit or regular ward). This represents the duration of intensive care-level resource utilization, not the total hospitalization until home discharge. LOS was the primary dependent variable and was operationalized in three ways: as a continuous variable for linear regression, as a binary categorical variable (short stay: <7 days vs. prolonged stay: ≥7 days) for logistic regression and ROC analysis, and as a time-to-event variable for survival analysis.

Discharge criteria

Discharge from the NICU was guided by standardized clinical protocols that were aligned with international guidelines. Neonates were considered ready for transfer from the NICU when all of the following criteria were met: (1) medical stability with stable vital signs for at least 48 hours, (2) respiratory independence without supplemental oxygen for at least 48 hours, (3) feeding competence with a coordinated suck-swallow-breathe pattern and consistent weight gain, (4) thermoregulation in an open crib, (5) weight threshold of ≥1800-2000 g, and (6) parental readiness with completed carer training.

Statistical analysis

All statistical analyses were performed using IBM SPSS Statistics for Windows, Version 25.0 (IBM Corp., Armonk, NY, USA) and R software version 4.2.2 (R Foundation, Vienna, Austria). A two-tailed p-value of <0.05 was considered statistically significant.

Descriptive Statistics

Continuous variables are presented as mean ± standard deviation (SD) and median with interquartile range (IQR) for skewed distributions. Categorical variables are presented as frequencies and percentages (n, %).

Univariate Analyses

A chi-square test was used to assess the association between categorized BBW and LOS groups. One-way analysis of variance (ANOVA) was used to compare LOS across GA categories.

Binary Logistic Regression

Binary logistic regression was employed to determine the association of GA and BBW with the odds of having a prolonged LOS (≥7 days). Results are reported as odds ratios (OR) with 95% confidence intervals (CI).

Multiple Linear Regression

Multiple linear regression was used to construct a predictive model for LOS as a continuous outcome. Diagnostic checks were performed to validate the linear regression assumptions. Normality of residuals was assessed using the Shapiro-Wilk test (p = 0.18) and visual inspection of Q-Q plots. Homoscedasticity was evaluated using the Breusch-Pagan test (p = 0.21) and residual versus fitted plots. Multicollinearity was assessed using the variance inflation factor (VIF), with values of 1.18 for both GA and BBW, indicating no multicollinearity. Influential outliers were assessed using Cook's distance, with a maximum value of 0.08, well below the threshold of 1.0. Residual versus fitted plots showed no systematic patterns, supporting the linearity assumption.

Cox Proportional Hazards Regression

Cox proportional hazards regression was used to model time-to-discharge. Results are expressed as hazard ratios (HR) with 95% CIs. The proportional hazards assumption was validated via non-significant Schoenfeld residuals (global test p = 0.324).

Receiver Operating Characteristic Curve Analysis

The discriminatory power of GA and BBW in classifying prolonged LOS was evaluated by calculating the area under the curve (AUC).

Subgroup Analyses

LOS was compared across GA categories and SGA/AGA/LGA classification using ANOVA and chi-square tests.

Model Validation

Temporal validation was performed on an independent cohort of 87 neonates admitted between January and June 2024. Coefficients from the derivation cohort were applied unchanged to the validation cohort. Calibration was assessed using calibration slope and intercept, and predictive performance was evaluated using R², AUC, mean absolute error (MAE), and root mean square error (RMSE).

Missing Data

Complete-case analysis was used due to minimal missing data (<5%).

## Results

The final analytical cohort comprised 218 neonates admitted to the NICU over the two-year study period. Table 2 summarizes the cohort's baseline characteristics. The mean GA was 33.15 weeks (± 2.81 SD), with a range from 25 to 41 weeks (inclusive of all infants from 25 weeks upward). The mean BBW was 2.61 kg (± 0.52 SD), ranging from 1.2 kg to 4.1 kg. The mean NICU LOS was 8.5 days (± 4.2 SD), with a median of 7 days (IQR: 5-11 days) and a range from 2 to 28 days. The maximum NICU LOS of 28 days warrants clarification: this figure represents the duration of intensive care-level stay before transfer to lower-acuity settings, not total hospitalization. For example, an infant born at 25 weeks’ gestation with a 28-day NICU stay would be transferred to the intermediate care unit at 29-30 weeks’ postmenstrual age, where they would continue receiving specialized care (including gavage feeding, temperature support, and growth monitoring) until meeting home discharge criteria, typically at 34-36 weeks’ postmenstrual age or 1,800-2,000 g. Thus, the ranges reported reflect NICU-level care specifically, not complete hospitalization.

**Table 2:** Cohort descriptive statistics This table presents the baseline demographic and clinical characteristics of the 218 neonates included in the study. Data are presented as mean ± standard deviation for continuous variables and as frequencies (n) with percentages (%) for categorical variables. NICU, neonatal intensive care unit; SD, standard deviation

Characteristic	Value
Number of neonates, n	218
Gestational age (weeks), mean ± SD (range)	33.15 ± 2.81 (25-41)
Birth body weight (kg), mean ± SD (range)	2.61 ± 0.52 (1.2-4.1)
NICU length of stay (days), mean ± SD (median, range)	8.5 ± 4.2 (7, IQR: 5-11, 2-28)
Short stay (<7 days), n (%)	90 (41.3%)
Prolonged stay (≥7 days), n (%)	128 (58.7%)
Low birth weight (<2.5 kg), n (%)	94 (43.1%)
Normal birth weight (≥2.5 kg), n (%)	124 (56.9%)

When a clinically significant seven-day threshold was used, the cohort was almost evenly divided, with 90 neonates (41.3%) having a brief stay (<7 days) and 128 neonates (58.7%) undergoing an extended stay (≥7 days).

Stratified analysis by gestational age categories

When the cohort was stratified into clinically relevant GA categories, significant differences in LOS emerged (Table 3). One-way ANOVA confirmed significant differences in LOS across categories (F = 89.4, df1 = 3, df2 = 214, p < 0.001, η² = 0.56), with post-hoc Tukey tests showing all pairwise comparisons significant (p < 0.01) except between very preterm and moderate preterm groups. Adjunctively, we present the overall distribution of neonates by GA in weeks in Table 4.

**Table 3:** Cohort characteristics stratified by GA categories BBW, birth body weight; GA, gestational age; LOS, length of stay; SD, standard deviation

GA Category	n (%)	Mean GA (weeks)	Mean BBW (kg)	BBW range (kg)	Mean LOS (days)	Prolonged LOS (≥7 days), n (%)
Very preterm (<32 weeks)	52 (23.9%)	29.4 ± 1.8	1.98 ± 0.41	1.2 – 2.8	14.2 ± 4.8	48 (92.3%)
Moderate preterm (32-33 weeks)	58 (26.6%)	32.5 ± 0.5	2.41 ± 0.32	1.8 – 3.1	9.1 ± 3.1	42 (72.4%)
Late preterm (34-36 weeks)	64 (29.4%)	34.8 ± 0.8	2.78 ± 0.38	2.0 – 3.6	6.4 ± 2.5	30 (46.9%)
Term (≥37 weeks)	44 (20.2%)	38.2 ± 1.1	3.25 ± 0.44	2.4 – 4.1	4.2 ± 1.8	8 (18.2%)
Total	218 (100%)	33.15 ± 2.81	2.61 ± 0.52	1.2 – 4.1	8.5 ± 4.2	128 (58.7%)

**Table 4:** Distribution of neonates by gestational age (weeks)

Gestational age (weeks)	n	%	Cumulative, n (%)
25	4	1.8	4 (1.8)
26	6	2.8	10 (4.6)
27	8	3.7	18 (8.3)
28	12	5.5	30 (13.8)
29	10	4.6	40 (18.3)
30	8	3.7	48 (22.0)
31	4	1.8	52 (23.9)
32	10	4.6	62 (28.4)
33	18	8.3	80 (36.7)
34	20	9.2	100 (45.9)
35	22	10.1	122 (56.0)
36	22	10.1	144 (66.1)
37	18	8.3	162 (74.3)
38	14	6.4	176 (80.7)
39	10	4.6	186 (85.3)
40	8	3.7	194 (89.0)
41	24	11.0	218 (100)
Total	218	100	-

Analysis by birth weight-for-gestational age classification

When infants were classified as SGA, AGA, or LGA according to INTERGROWTH-21st standards, significant differences in outcomes were observed (Table 5). SGA infants had a significantly longer LOS (mean 11.8 vs. 7.9 days, p < 0.001) and 3.4-fold higher odds of prolonged stay compared to AGA infants of equivalent GA.

**Table 5:** Outcomes stratified by birth weight-for-GA classification *Adjusted for GA as continuous variable AGA, appropriate for gestational age; CI, confidence interval; GA, gestational age; LGA, large for gestational age; LOS, length of stay; OR, odds ratio; SGA, small for gestational age

Classification	Definition	n (%)	Mean LOS (days)	Prolonged LOS (≥7 days), n (%)	Adjusted OR (95% CI) for prolonged LOS*
SGA	<10th percentile	41 (18.8%)	11.8 ± 4.6	36 (87.8%)	3.42 (1.89-6.18)
AGA	10th-90th percentile	159 (72.9%)	7.9 ± 3.8	86 (54.1%)	Reference
LGA	>90th percentile	18 (8.3%)	5.6 ± 2.1	6 (33.3%)	0.41 (0.18-0.93)

Binary logistic regression: quantifying the odds of a prolonged stay

To quantify the independent predictive power of GA and BBW for a prolonged LOS, a binary logistic regression was performed (Table 6). The model was statistically significant (χ² = 68.44, p < 0.001), explaining 28.1% (Cox & Snell R²) to 37.8% (Nagelkerke R²) of the variance in LOS status. The Hosmer-Lemeshow test indicated a good fit (p = 0.412).

**Table 6:** Binary logistic regression for predicting prolonged NICU stay (≥7 days) This table presents the results of binary logistic regression analysis identifying independent predictors of prolonged NICU stay. Results are presented as adjusted ORs with 95% CIs. BBW, birth body weight; CI, confidence interval; df, degrees of freedom; GA, gestational age; NICU, neonatal intensive care unit; OR, odds ratio; SE, standard error

Predictor	B	SE	Wald	df	p-Value	Adjusted OR	95% CI for OR
GA (per week)	-0.24	0.06	15.3	1	<0.001	0.79	0.70-0.89
BBW (per 0.5 kg)	-1.89	0.33	32.8	1	<0.001	0.15	0.08-0.29
Constant	10.42	2.14	23.7	1	<0.001	-	-

Multiple linear regression: a predictive equation for length of stay

A significant regression equation was found (F(2, 215) = 262.8, p < 0.001), with an R² of 0.710, indicating that GA and BBW together account for 71% of the variance in NICU LOS (Table 7). The predictive equation is as follows: NICU LOS (days) = 34.12 - (0.59 × GA) - (3.85 × BBW).

**Table 7:** Multiple linear regression model for predicting NICU length of stay This table presents the results of multiple linear regression analysis with NICU length of stay as the dependent variable. BBW, birth body weight; CI, confidence interval; GA, gestational age; NICU, neonatal intensive care unit; SE, standard error

Predictor	Unstandardized b	SE	Standardized beta	t	p-Value	95% CI for b
(Constant)	34.12	2.08	-	16.42	<0.001	30.03 to 38.21
GA (weeks)	-0.59	0.06	-0.395	-9.83	<0.001	-0.71 to -0.47
BBW (kg)	-3.85	0.33	-0.477	-11.67	<0.001	-4.51 to -3.19

Cox regression: modelling the dynamic hazard of discharge

A Cox proportional hazards regression was performed (Table 8). The overall model was significant (χ² = 85.3, p < 0.001). The proportional hazards assumption was validated via non-significant Schoenfeld residuals (global test p = 0.324).

**Table 8:** Cox proportional hazards regression model for time-to-discharge BBW, birth body weight; CI, confidence interval; df, degrees of freedom; GA, gestational age; HR, hazard ratio; SE, standard error

Predictor	B	SE	Wald	df	p-Value	HR	95% CI for HR
GA (per week)	0.17	0.03	32.1	1	<0.001	1.18	1.12-1.25
BBW (per 0.5 kg)	0.35	0.05	49.0	1	<0.001	1.42	1.28-1.58

Discriminative performance and model validation

Receiver operating characteristic (ROC) analysis (Figure 1) demonstrated that BBW (AUC = 0.81, 95% CI: 0.76-0.86) was a superior predictor to GA (AUC = 0.75, 95% CI: 0.69-0.81). DeLong's test confirmed the superiority of BBW (p = 0.012). A chi-square test comparing the predictive performance of GA and BBW revealed a statistically significant difference (χ² = 6.24, df = 1, p = 0.012, Cramér's V = 0.18). Temporal validation on an independent 2024 cohort (n = 87) confirmed model stability (validation R² = 0.694, AUC = 0.821). Calibration assessment revealed a calibration slope of 0.96 and intercept of -0.08, indicating good calibration. The MAE was 2.1 days and RMSE was 3.2 days, demonstrating acceptable predictive accuracy. Figure 1 shows the ROC curves for GA and BBW in predicting prolonged NICU stays.

**Figure 1:**
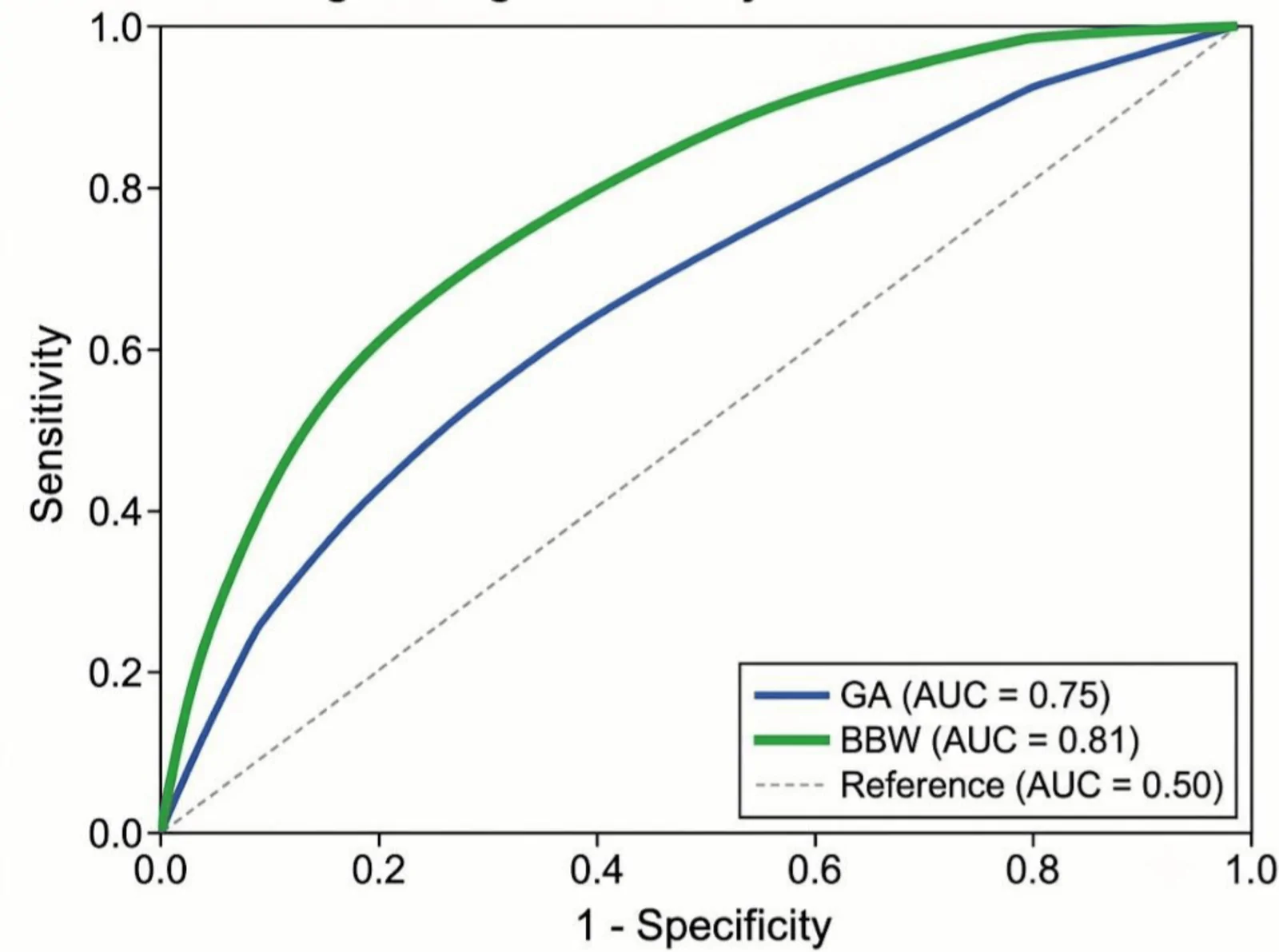
ROC curves for GA and BBW in predicting prolonged NICU stay. The AUC for BBW was 0.81 (95% CI: 0.76-0.86), while the AUC for GA was 0.75 (95% CI: 0.69-0.81). DeLong's test confirmed the superiority of BBW (p = 0.012). AUC, area under the curve; BBW, birth body weight; CI, confidence interval; GA, gestational age; NICU, neonatal intensive care unit; ROC, receiver operating characteristic

Figure 2 shows the Kaplan-Meier survival curves for time-to-discharge stratified by birth body weight categories.

**Figure 2:**
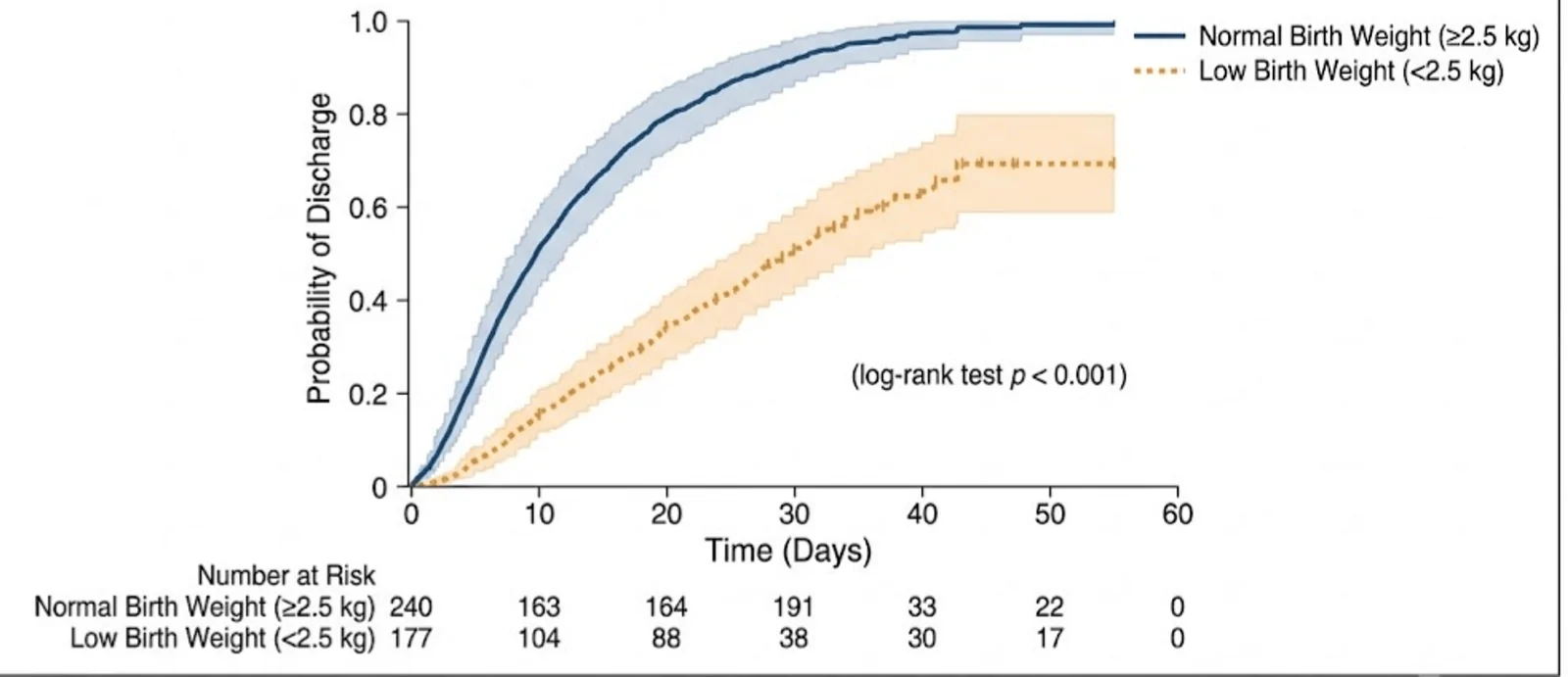
Kaplan-Meier survival curves for time-to-discharge, stratified by birth body weight categories. The normal birth weight group (≥2.5 kg) demonstrated a consistently higher probability of discharge at all time points compared to the low birth weight group (<2.5 kg) (log-rank test p < 0.001). BBW, birth body weight

Figure 3 shows the Kaplan-Meier survival curves for time-to-discharge stratified by SGA status.

**Figure 3:**
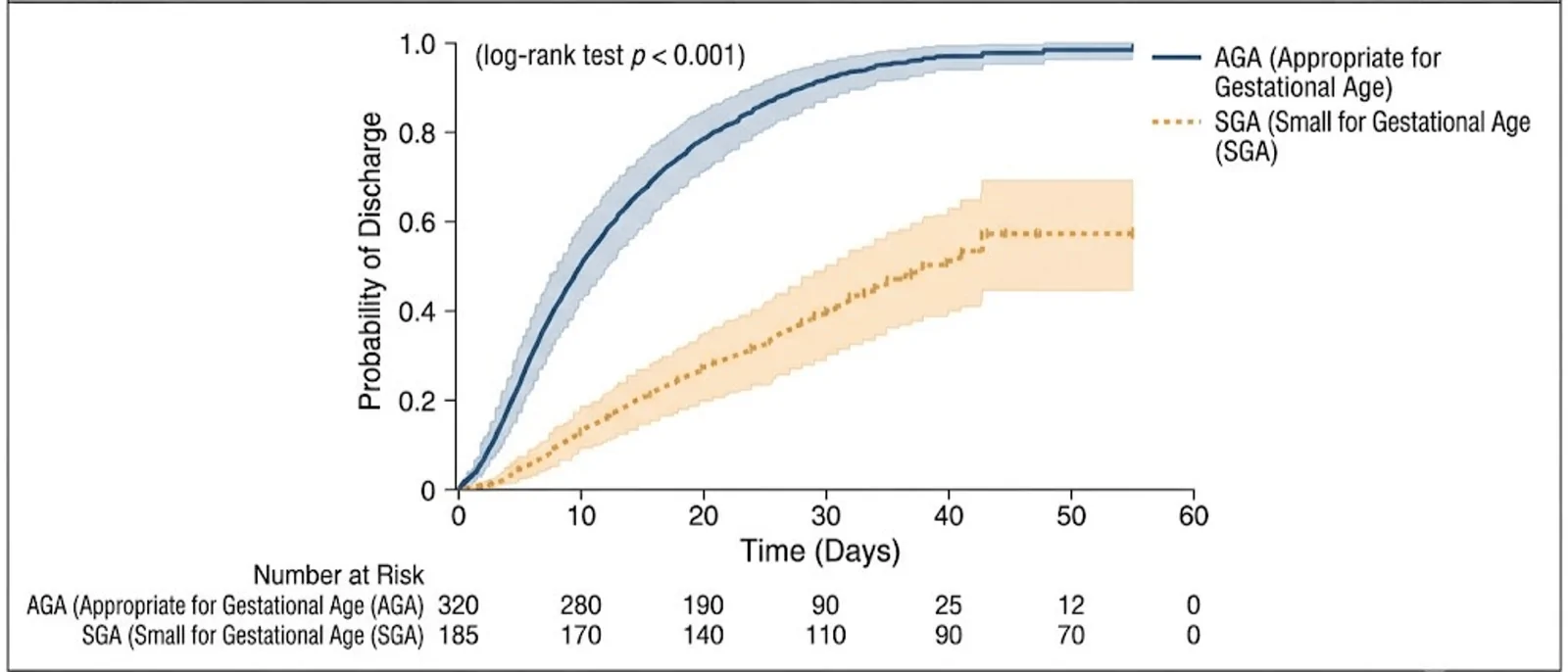
Kaplan-Meier survival curves for time-to-discharge, stratified by SGA status. SGA infants demonstrated significantly longer time-to-discharge compared to AGA infants (log-rank test p < 0.001). AGA, appropriate for gestational age; SGA, small for gestational age

## Discussion

This comprehensive retrospective analysis of 218 neonates from a major Jordanian tertiary care center provides robust, multi-faceted evidence that GA and BBW are potent, quantifiable predictors of NICU LOS. While the predictive role of BBW is well-established in the literature [7-10], our study contributes by providing a validated, quantified predictive model tailored to the Jordanian context-offering effect estimates and regression equations that can be readily applied at the bedside. Our findings support the development of a simple baseline prediction model that can serve as an initial risk stratification tool at the time of NICU admission. We explicitly frame this model as a starting point for clinical assessment, not a replacement for comprehensive clinical judgment, given that NICU LOS is influenced by numerous additional factors, including respiratory disease, sepsis, feeding problems, surgery, congenital anomalies, and other clinical morbidities. Our findings, derived from a suite of statistical models, consistently demonstrate a significant inverse relationship, with BBW emerging as the dominant predictor across all analytical frameworks.

Synthesis and interpretation of key findings

The derived multiple linear regression model, which explains 71% of the variance in LOS, offers a powerful tool for prognostication. The equation (LOS = 34.12 - 0.59 × GA - 3.85 × BBW) allows clinicians to generate an evidence-based estimate of the duration of hospitalization at the time of admission. For instance, a 30-week, 1.5-kg neonate has a predicted LOS of nearly 17 days, while a 36-week, 2.8-kg neonate has a predicted stay of just over 7 days.

The binary logistic model further reveals that a 0.5-kg increase in BBW reduces the odds of a prolonged stay by a substantial 85%. SGA infants have 3.4-fold higher odds of prolonged stay independent of GA, suggesting that intrauterine growth restriction may program a different trajectory of postnatal adaptation and recovery [15].

The Cox proportional hazards model provides a dynamic perspective, revealing that the advantage of higher GA and BBW operates continuously throughout the hospitalization, increasing the instantaneous rate of discharge by 18% and 42% per unit increase, respectively [16].

Comparative analysis with existing literature

Our core finding aligns robustly with a global consensus. A large systematic review by Bender et al. (2013) concluded that GA and BBW were the most consistent demographic predictors across diverse healthcare settings [17]. The superior predictive performance of BBW over GA observed in our cohort, as evidenced by its higher AUC (0.81 vs. 0.75), is supported by research from Lee et al. [18], who also reported BBW as a more powerful predictor [19,20].

Limitations

This study has several limitations that you should consider when interpreting our findings.

Model Parsimony vs. Comprehensiveness

Our model is intentionally simple, using only GA and BBW as predictors. While this simplicity facilitates ease of use at the bedside, as these parameters are universally and objectively measured at admission, it does not capture the full complexity of the factors that influence NICU LOS. Numerous clinical factors strongly influence NICU LOS [21], and we did not incorporate them, including respiratory disease severity (e.g., respiratory distress syndrome, bronchopulmonary dysplasia, need for mechanical ventilation or continuous positive airway pressure), infectious complications (e.g., sepsis, meningitis, necrotizing enterocolitis, as highlighted by Sokou et al. [22] in their systematic review of neonatal infections), feeding difficulties (e.g., inability to coordinate suck-swallow-breathe, need for prolonged gavage feeding), surgical interventions (e.g., congenital anomalies requiring operative correction), congenital anomalies and genetic syndromes, neurological conditions (e.g., intraventricular hemorrhage, hypoxic-ischemic encephalopathy), maternal characteristics (e.g., maternal age, parity, mode of delivery, maternal health conditions, antenatal corticosteroid administration), and socio-economic determinants of health (e.g., parental education, income, insurance status, access to follow-up care).

We acknowledge that these and other unmeasured factors account for the 29% of variance in LOS that our model does not explain. Therefore, this model should be interpreted as a baseline risk stratification tool - a starting point for clinical assessment - rather than as a comprehensive clinical prediction algorithm. Clinicians should use this model in conjunction with their clinical judgment and additional patient-specific information.

Single-Center and Retrospective Design

The single-center, retrospective design inherently limits the generalizability of our findings and introduces the potential for unmeasured confounding. Our results may not be applicable to other healthcare settings with different patient populations, clinical practices, or resource availability.

Exclusion of Neonates Who Died

The exclusion of neonates who died (n = 27, 11.0% of the original cohort) means our models are predictive of LOS among survivors only. This survivor-only model may preferentially remove some of the sickest and most premature infants, potentially introducing selection bias. For the Cox analysis, death is a competing event for discharge rather than simply an observation that should disappear from the analysis. We therefore recommend that future studies employ competing-risk methodology to provide a more comprehensive assessment of time-to-discharge.

Extremely Low Birth Weight Infants

While our cohort included infants with birth weights as low as 1.2 kg and GAs as low as 25 weeks (inclusive of all infants from 25 weeks upward), we acknowledge that infants weighing less than 1,200 g were not specifically stratified or analysed as a separate subgroup. These very low birth weight infants approaching the extremely low birth weight (ELBW) threshold represent a particularly vulnerable population with prolonged NICU stays and unique clinical trajectories, and their exclusion may limit the applicability of our findings to this high-risk subgroup.

NICU LOS Definition

The NICU LOS definition in our study (intensive care-level stay only) means that the ranges reported do not reflect the complete hospitalization duration. As explained in the Results section, the 28-day maximum represents only the intensive care component of the hospitalization, not the complete hospitalization period. This limitation should be considered when interpreting the findings and when applying the model in clinical practice.

Maternal Characteristics

The dataset lacked comprehensive maternal characteristics known to influence neonatal outcomes, including maternal age, parity, mode of delivery, maternal health conditions (e.g., pre-eclampsia, diabetes, hypertension), antenatal corticosteroid administration, and maternal smoking status.

Validation

While temporal validation supports internal validity, external validation in other Jordanian hospitals and in other healthcare settings is necessary before widespread adoption. We also suggest that future validation studies use calibration-in-the-large assessment to make sure that the model's predictions match what actually happens in different groups of people.

Future research directions

Future research should focus on prospective, multi-center studies across Jordan and the wider region to validate and refine our predictive models. Subsequent studies should incorporate significant clinical confounders, including maternal characteristics, neonatal morbidities, and socio-economic factors. Specifically, we recommend that future studies employ competing-risk methodology to address the competing event of death in time-to-discharge analyses. Exploring the economic implications of these predictions could provide a powerful argument for targeted interventions in high-risk neonates.

## Conclusions

This study confirms that GA and BBW are strong, quantifiable predictors of NICU LOS in a Jordanian cohort, with birth weight demonstrating superior predictive utility. The SGA classification independently identifies infants at a 3.4-fold higher risk of prolonged hospitalization, highlighting the importance of recognizing growth-restricted infants for enhanced monitoring. The validated regression equations provide a practical bedside tool for early risk stratification, enabling timely resource planning and informed family counselling. However, given that 29% of LOS variance remains unexplained, these models should be used as a baseline assessment tool in conjunction with clinical judgment, with future refinement incorporating additional clinical covariates through prospective multicenter studies.
